# Peer review for biomedical publications: we can improve the system

**DOI:** 10.1186/s12916-014-0179-1

**Published:** 2014-09-26

**Authors:** Philip F Stahel, Ernest E Moore

**Affiliations:** Department of Orthopaedics, University of Colorado, School of Medicine, Denver Health Medical Center, 777 Bannock Street, Denver, CO 80204 USA; Department of Neurosurgery, University of Colorado, School of Medicine, Denver Health Medical Center, 777 Bannock Street, Denver, CO 80204 USA; Department of Surgery, University of Colorado, School of Medicine, Denver Health Medical Center, 777 Bannock Street, Denver, CO 80204 USA

**Keywords:** Peer review, Biomedical publications, Evidence-based medicine, Randomized controlled trials

## Abstract

The lack of formal training programs for peer reviewers places the scientific quality of biomedical publications at risk, as the introduction of ‘hidden’ bias may not be easily recognized by the reader. The exponential increase in the number of manuscripts submitted for publication worldwide, estimated in the millions annually, overburdens the capability of available qualified referees. Indeed, the workload imposed on individual reviewers appears to be reaching a ‘breaking point’ that may no longer be sustainable. Some journals have made efforts to improve peer review via structured guidelines, courses for referees, and employing biostatisticians to ensure appropriate study design and analyses. Further strategies designed to incentivize and reward peer review work include journals providing continuing medical education (CME) credits to individual referees by defined criteria for timely and high-quality evaluations. Alternative options to supplement the current peer review process consist of ‘post-publication peer review,’ ‘decoupled peer review,’ ‘collaborative peer review,’ and ‘portable peer review’. This article outlines the shortcomings and flaws in the current peer review system and discusses new innovative options on the horizon.

See related article: http://www.biomedcentral.com/content/pdf/s12916-014-0128-z.pdf.

**Electronic supplementary material:**

The online version of this article (doi:10.1186/s12916-014-0179-1) contains supplementary material, which is available to authorized users.

## Background

We read with enthusiasm the recent opinion article by Jigisha Patel in *BMC Medicine* [1]. Dr. Patel provides a critical analysis of the shortcomings and ‘hidden dangers’ of the established peer review process in biomedical publishing, with a focus on peer review for randomized controlled trials (RCTs) [1]. The lack of coherent training and specialization of peer reviewers appears to jeopardize the scientific quality of published manuscripts [2]. Once published, articles of ‘hidden’ substandard quality will negatively affect the relevance of meta-analyses, clinical guidelines and evidence-based treatment recommendations (“Garbage in, garbage out!”) [3]. This notion is coherently illustrated by a quote from Dr. Patel’s current article:

*“Treatment decisions are based on evidence which is itself determined by a system for which there is no evidence of effectiveness”* [1].

### The peer review process ‘left behind’

Although the quality of evidence-based medicine (EBM) has evolved over the years with the provision of defined uniform criteria for reporting of trials (Consolidated Standards of Reporting Trials (CONSORT) statement [4]) and of meta-analyses (Quality of Reporting of Meta-analyses (QUOROM) statement [5]), we have not observed a similar evolution of the peer review process, and the current modalities of peer review warrant reconsideration. This is analogous to considering a modern 21st century information technology company running its operations on first-generation 4 kB Apple computers from 1976.

The exponential increase in the number of manuscripts submitted for publication worldwide overburden the capability of available qualified referees to keep up with reviewing requests and to ensure timeliness and quality of their respective evaluations. In 2006, the estimated number of published peer-reviewed articles reached 1.4 million per year [6]. As the rejection rate for average journals ranges 20 to 50% (and much higher for more prestigious journals), the number of submitted manuscripts undergoing a formal peer review is more likely to approach 2 to 3 million per year. These conservative estimates, originating from 2006, must be adjusted to the current time, based on the dramatic ‘inflation’ of new open-access journals sprouting like mushrooms all over the globe.

The ever-increasing competitiveness in research (“publish or perish”) in these current times of limited grant funding opportunities incentivizes researchers to ‘fragment’ results from a single study into multiple publications, or to publish identical data sets redundantly [7]. This effect contributes to the ever-increasing ‘flood’ of biomedical manuscripts submitted for publication globally.

### The burden on reviewers

The burden placed on peer reviewers to assess an increasing number of submitted manuscripts – a large proportion of which are characterized by questionable scientific quality – appears to be reaching a ‘breaking point’ that is no longer sustainable. Increasing numbers of reports on unethical research conduct, including the publication of fraudulent and fabricated data and of plagiarized or redundant publications, represent an additional dilemma for editors and reviewers [7-9]. Selected papers that are officially retracted tend to receive wide public attention [10,11]; however, such reports are likely to represent just the ‘tip of the iceberg’ of an unrecognized problem for the scientific community. Indeed, a highly provocative interpretation of biomedical publications claimed that most published research findings are misleading, and the result of an unjustified “chase for statistical significance” [12].

This raises the following questions:

- How are peer reviewers supposed to cope with the sheer number of increasing reviewing requests and assignments?
- How are untrained ‘lay’ referees expected to recognize and scrutinize flaws in study design, methodology, and the validity of interpretation of data?
- How are qualified ‘expert’ referees expected to recognize research misconduct and to stratify apparent ‘good papers’ from unethical submissions, including redundant publications and fabricated data?

### The burden on editors

As editors of two peer-reviewed journals, representing both a model of open-access (*Patient Safety in Surgery* [13]) and a traditional print journal (*Journal of Trauma and Acute Care Surgery* [14]), we are exposed to the daily challenge of identifying and commissioning suitable referees who are willing to accept a requested assignment and to return a quality report in a timely fashion. Indeed, ensuring a streamlined, fast-track, and high-quality peer review process remains the ultimate editorial responsibility and duty for the scientific community. Any flaw in the peer review process of submitted manuscripts will ultimately jeopardize the quality of evidence-based recommendations, which rely on the assumption that the quality of the published science should be impeccable.

Extrapolated to the court of law, would anybody accept a verdict from poorly qualified judges, purely based on the notion that those individuals were available to complete the assigned task? Clearly, the editorial process is highly responsible and challenging. Most editors spend a significant amount of time investigating the suitability of potential reviewers by matching their publication record to the topic of interest, and cross-checking potential referees for co-authorships with submitting authors. An editor’s ‘favorite’ type of reviewer comprises candidates who are immediately agreeable to accept requested assignments and who return a high-quality and comprehensive evaluation before expiration of the deadline. Despite diligent scrutiny to the process, as editors, we frequently remain uncertain as to the true qualifications of the assigned individual referees.

### The ‘ideal’ peer reviewer

In a perfect world, the ideal peer reviewer should constitute an active scientist working in the same subspecialty ‘niche’ of research matching the topic of the submitted paper, but should not have any current collaboration or professional liaison with the submitting authors, in order to avoid a conflict of interest. On the other hand, such expert ‘peers’ may easily be direct competitors for grant awards in the same field of research. This bias could provide the root cause of unjustified adverse reports leading to rejection of a submitted paper, or to a significant delay in publication by requesting additional cumbersome experiments. This type of ‘hidden’ conflict of interest may not be detectable by a journal’s managing editors.

### Flaws and fraud in the system

Recent worrisome reports describe a new pattern of peer review fraud, by which submitting authors falsify the contact information of suggested referees, with the goal of diverting the peer review request to their own email account under a falsified name. A recent report in the *New York Times* described a peer review fraud scheme run by a researcher in Taiwan, which led to a journal’s retraction of 60 publications [15]. The uncovered operation was designated as a “peer review and citation ring” consisting of fake researchers and real ones whose identity was assumed by the author, who created 130 fraudulent e-mail accounts used in the forged peer review process [15]. As most biomedical journals rely on an online submission and review system to assess submitted manuscripts, the ‘gray zone’ of online peer review fraud may be higher than assumed.

In light of all the shortcomings related to the current peer review process and its impact on the quality and practice of EBM, many critical voices have questioned the validity and sustainability of our current approach to scientific publishing [2,3,16,17]. A provocative recommendation by the forefront science group “The Edge” suggests completely abolishing EBM *per se* as an outdated scientific tenet, in answer to the annual question of 2014 *“What scientific idea is ready for retirement?”* [18,19].

### ‘Journal survival’ versus rigorous peer review

A recent in-house editorial analysis in 2012 to 2013 on the ‘fate’ of rejected manuscripts with the *Journal of Trauma and Acute Care Surgery* revealed that 42% of rejected papers were readily published in open-access journals within an average of 10 months after rejection (Crebs and Moore; unpublished observations). The interpretation of this finding is ambiguous. On the one hand, it is very possible that the scrutiny of the initial peer review process will help improve the overall quality of a rejected paper after revision, and thus make it more appealing and suitable for publication in a second-tier target journal. On the other hand, some open-access online journals appear to commission articles by a purely business incentive, without tribute to scientific merit and quality of research.

Provocatively speaking, many of the new generation open-access journals may tend to accept a lower threshold of peer review quality, or imply that in-house editorial decision-making is reflective of formal ‘peer review’ as a trade-off to sustain their financial viability [20]. This is particularly important as the revenue stream in the ‘author pays’ model is dependent on the high publication fee ($2,000 or more) charged to authors upon acceptance of their article for publication. For this reason, many scientists consider open-access peer review in general as intrinsically biased. A journal’s overall rejection rate may serve as a proxy or surrogate marker to the quality of peer review, in conjunction with the number of peer review cycles, the number of referees assigned to an individual manuscript, and the commissioning of re-reviews and application of editorial changes prior to acceptance. These metrics could be transparently incorporated in a peer review ‘quality mark’ included in each publication, as suggested in Dr. Patel’s article [1].

### New models on the horizon

Despite the negative headlines and acknowledged deficiencies in the system, there have been significant efforts to improve the quality of the current modality of biomedical peer review. For example, the *Journal of Trauma and Acute Care Surgery* 1) selects reviewers based on their publication record; 2) assigns reviewers based on a list of those considered experts in the topic; 3) provides continuing medical education (CME) credits for high-quality reviews and timeliness of completion; 4) provides formal annual education sessions on how to conduct peer review; and 5) employs a MD/PhD biostatistician to review all provisionally accepted manuscripts. The *Journal* furthermore provides uniform guidelines for reviewers (see Additional file 1: Appendix 1) which appear particularly helpful for younger and less experienced scientists at an early stage of their career. Other journals, including the *Journal of Bone and Joint Surgery*, recently adopted a new grading system for the quality of peer review, termed “peer review evaluation” (PRE) score, which is based on defined objective metrics including the overall number of review cycles. PRE score is designed to measure the level of quality of peer review under the assumption that a more engaged peer review process will result in a higher quality final publication. Additional new concepts that have been recently advocated as alternatives include ‘post-publication peer review’ , ‘collaborative peer review’ , and ‘decoupled peer review’ [1]. Finally, third-party evaluations managed by for-profit companies have recently been offered as an independent ‘portable peer review’ , paid for by the author and moved between journals until a final editorial decision is made [21].

## Conclusion

In summary, we applaud Dr. Patel’s important contribution, which identifies the multiple shortcomings of the current peer review process for biomedical publishing, and offers specific pertinent solutions to improve the system [1]. It is ultimately our duty as editors and scientists to move the field forward, as we can no longer accept the standard excuse of peer review being a “broken system – but still the best we have”. We can improve the system.

## Additional file

Additional file 1: Appendix 1. Journal of Trauma and Acute Care Surgery – Guidelines for Peer Reviewers.
