# Supplementary material for: Peer review for biomedical publications: we can improve the system
Source: BMC Med. 2014 Sep 26;12:179. doi: 10.1186/s12916-014-0179-1 (PMC4177268; doi:10.1186/s12916-014-0179-1)
Supplement: Additional file 1: Appendix 1. — Journal of Trauma and Acute Care Surgery – Guidelines for Peer Reviewers. [file 12916_2014_179_MOESM1_ESM.pdf]

Journal of Trauma and Acute Care Surgery

Editor: Ernest E. Moore, M.D.

Associate Editors: David B. Hoyt, M.D., Ronald V. Maier, M.D., and Steven R. Shackford, M.D.

GUIDELINES FOR REVIEWERS

PEER REVIEW AND DISCLOSURE

All original material presented in the *Journal of Trauma and Acute Care Surgery* undergoes rigorous assessment by knowledgeable and dedicated reviewers who are recognized as leaders in their respective domains.

Although historically only authors have been required to disclose financial or personal interests that may bias their presentation of research, the *Journal* now requires disclosure of those involved in the review process. To that end, accepted reviewers will be asked to disclose any conflicts of interest prior to submitting a review.

GENERAL GUIDELINES

- Unpublished manuscripts under review are privileged and confidential documents. Reviewers are expected to protect manuscripts from any form of exploitation, to refrain from citing a manuscript or the work it describes before publication, and to not use the data it contains for the advancement of their own research agenda.

- The ideal reviewer consciously adopts an impartial attitude toward the manuscript under review. Reviewers should strive to be an author's ally, with the aim of facilitating effective and accurate scientific communication.

- If you are able to review, please accept the assignment **within 3 days**. If we do not hear from you within that time, we will proceed with an alternate reviewer.

- If you believe that you cannot judge a given article impartially or complete a review within the given timeframe, please follow the login instructions and select 'Decline to Review' as soon as possible. In the response field, please include the following:
    - A reason for declining to review the manuscript.
    - Suggested colleague(s) qualified to review this paper.

- Reviews should be completed **within two weeks** (14 days from acceptance of assignment). If you have already accepted an assignment, but know that you cannot finish the review within that time, please contact the Editorial Office at (303) 602-1815 to determine what action should be taken.

Assessing the Manuscript

In an effort to standardize the review process for the *Journal of Trauma and Acute Care Surgery*, we ask that you consider the following questions when assessing a manuscript for possible publication:

Why was the study done?
Does it address either an important unsolved problem of clinical relevance or a basic scientific topic relevant to trauma and acute care surgery? Do you think that there is sufficient evidence to justify the study? Have the authors explicitly stated a study purpose or a hypothesis?

How was the study done?
What is the design and is it explicitly stated by the authors in the methods?

Is the study population defined well?
Do the authors explicitly define inclusion and exclusion criteria? Are all of the patients accounted for in the results section?

Are the outcome measures appropriate?
Are the selected variables suitable to the study purpose or hypothesis? Are confounding variables assessed?

Are the analytical methods appropriate?
Was the hypothesis sufficiently tested? Were appropriate statistical analyses or laboratory diagnostics performed? Was a power analysis done?

What is the significance of the work?
Does the study present novel results that will add to the literature? Are previous similar studies discussed? Are potential study limitations addressed? Are the conclusions warranted by the data?

Planning your Review

Please be prepared to comment on the following aspects of the manuscript, as far as they are applicable, in your review:

- Overall novelty/interest of the research question
- Coherence and completeness of the background
- Clarity of hypothesis or study objectives
- Adequacy of methods or experimental approach
- Soundness of data interpretation and conclusions
- Clarity of writing, strength and organization of the paper
- Relevance, accuracy and completeness of bibliography
- Number and quality of figures, tables and illustrations

Getting Started

Before filing comments, you will be asked several preliminary questions. These include:

- Do you have any conflicts of interest relating to this manuscript?
- Do you agree with the authors' level of evidence rating for this study?
- Do you have reason to believe that this manuscript (in whole or part) has published before?
- Would you be willing to write an editorial critique to appear with this paper, if accepted?
- Should this manuscript be reviewed by a biostatistician?

CME Credit

Reviewers for the *Journal of Trauma* may earn CME credit for completing reviews.

Once all requested reviews are filed and a final decision is made, the editor will grade the quality of your review. CME credit will be awarded if your review is found to be timely and constructive, regardless of your decision. Certificates are generally emailed within 2 months of a final decision.

To be eligible to earn CME credit, you will need to answer the following four questions:

- Are you interested in earning continuing education credit? (*AMA PRA Category 1 Credit*™)
- How long did it take to complete this review?
- Performing this review has improved my knowledge and ability to assess the scientific literature in order to make informed decisions in my practice.
- Performing this review has improved my critical thinking and writing skills within my area of expertise.

The editor will evaluate your review and assign a score between 0 and 100 to reflect the quality of the review. A score of 70 or above is needed in order to earn CME credit. (Please note that this evaluation of your review is distinct from the quality of the article. Credit will be awarded if your review is thorough and constructive, regardless of your decision term)

At the end of each month, our publisher's Continuing Education Department personnel will download a report from Editorial Manager that contains your responses and the editor's scoring. For eligible reviews, the publisher's CME Department will email a certificate to the reviewer. In accordance with provider guidelines, physicians (MDs and DOs) will earn up to 3 *AMA PRA Category 1 Credit*™ credits commensurate with the amount of time spent doing the review.

Questions?

For more information about editorial criteria for CME-eligible reviews, contact the editorial office anytime (+1 303-602-1815 or +1 303-602-1816). If you do not receive your certificate after two months, call LWW's Continuing Education Department for more information (+1 215-521-8636).

LEVELS OF EVIDENCE

The Journal requires authors of clinically-oriented studies to indicate a Level of Evidence and study type at the end of their abstract. **Please note that only clinical studies receive levels of evidence; basic science, animal studies, reviews, etc. do not require Levels of Evidence.**

To quickly determine the level under which a study falls, please consult the following table:

Evidence Levels for Individual Studies (*J Trauma Acute Care Surg.* 2012;72(6):1484-90)

	Therapeutic / Care Management	Prognostic and Epidemiological	Diagnostic Tests or Criteria	Economic & Value-based Evaluations	Systematic Reviews & Meta-analyses
**Level I**	RCT with no negative criteria*	Prospective† study with large effect‡ and no negative criteria*	Testing of previously developed diagnostic criteria in consecutive patients (all compared to "gold" standard) and no negative criteria.	Sensible costs and alternatives; values obtained from many sources; multi-way sensitivity analyses	Systematic Review (SR) or meta-analysis (MA) of predominantly level I studies and no SR/MA negative criteria †
**Level II**	• RCT with significant difference and only one negative criterion*<br>• Prospective† comparative study without negative criteria*<br>• Prospective/retrospective† study with large effect‡ and only one negative criterion*	• Prospective† study with less than large effect‡ and no negative criteria*<br>• Untreated controls from RCT	Development of diagnostic criteria on consecutive patients (all compared to "gold" standard) and only one negative criterion.	Sensible costs and alternatives; values obtained from limited sources; multi-way sensitivity analyses	SR / MA of predominantly level II studies with no SR/MA negative criteria †
**Level III**	• Case-control study without negative criteria*<br>• Prospective† comparative study with only one negative criterion*<br>• Retrospective† comparative study without negative criteria*	• Case-control study without negative criteria *<br>• Prospective/retrospective† study with up to two negative criteria*	Nonconsecutive patients (without consistently applied "gold" standard) with up to two negative criteria.	Analyses based on limited alternatives and costs; poor estimates	SR /MA with up to two negative criteria †
**Level IV**	Prospective/retrospective† study using historical controls or having more than one negative criterion*	Prospective/retrospective† study with up to three negative criteria*	Case-control study with no negative criteria* or other designs with up to three negative criteria.	No sensitivity analyses	SR/MA with more than two negative criteria †
**Level V**	• Case series<br>• Studies with quality worse than level IV	• Case series<br>• Studies with quality worse than level IV	No or poor "gold" standard		

* Negative criteria decreasing level of evidence include: (1) <80% follow-up; (2) >20% missing data or missing data not at random without proper use of missing data statistical techniques; (3) limited control of confounding (e.g., mortality comparisons with inadequate risk adjustment); (4) more than minimal bias (selection bias, publication bias, report bias, etc.); (5) heterogeneous populations (e.g., institutions with distinct protocols/patient volume, conditions caused by distinct pathogenic mechanisms); and (6) for RCT only, no blinding or improper randomization; (7) Inadequate statistical power: this only applies to studies NOT finding statistical differences and it is defined as power <80% for declaring "failure to detect a significant difference" or power <90% for declaring "bio-equivalence or non-inferiority or comparative effectiveness" or Receiver Operating Characteristic curve <80% or both sensitivity and specificity <80%.

†Prospective versus retrospective: studies with data collected to answer predefined questions are prospective; studies with data collected for questions unrelated to the original question for which the data were gathered are retrospective.

‡Large effect is defined as: (1) study with large RR (95 or 0.2) about condition of low-to-moderate morbidity/mortality and (2) study with moderate-to-large RR (2Y5 or 0.2Y0.5) about condition of high morbidity/mortality. Large effect includes the following: (1) study with large RR (95 or G0.2) about condition of low-to-moderate morbidity/mortality and (2) study with moderate-to-large RR (>5 or <0.2) about condition of high morbidity/mortality. §Negative criteria for SR/MS (decreases level of evidence): (1) no or inadequate standard search protocol, (2) more than minor chance of publication bias or publication bias not assessed, (3) moderate heterogeneity of included studies and/or populations (e.g., elective operation and acute operation), (4) predominance of level III or lower studies, and (5) no measures or inappropriate measures of pooled risk (for meta-analysis only).

‖ Adequate statistical power: this only applies to studies not finding statistical differences, and it is defined as power 980% for declaring "failure to detect a significant difference" or power 990% for declaring "bioequivalence or noninferiority or comparative effectiveness."

In addition to the level, studies will receive a + to designate whether standard reporting format was followed (e.g., CONSORT for RCTs). Authors can find reporting guidelines for most studies at the international EQUATOR Network.

Reviewer Checklist

Conflict of Interest

- ❑ Ensure and indicate that you have no conflict(s) of interest in reviewing the paper.

Abstract and Introduction

- ❑ Abstract is concise and structured (containing subheads for Background, Materials/Methods, Results, Conclusions, and Levels of Evidence).
- ❑ Abstract does not cite references.
- ❑ Abstract includes three to five keywords.
- ❑ Introduction concludes with specific hypothesis or stated goal of the study.
- ❑ Abbreviations are defined at first mention in text and in each table and figure.

Materials and Methods

- ❑ The clinical population or laboratory model to be discussed is described and justified concisely.
- ❑ Experimental design permits appropriate statistical assessment and ensures that the question(s) being asked can be answered.
- ❑ In longitudinal clinical studies, the patients are stratified by year and studied to account for changes in clinical care that occur over time.
- ❑ All variables that may influence findings are controlled (as far as possible).
- ❑ Variables of interest are listed, assay procedures are described, and scientific devices are identified.
- ❑ Statistical assays are pre-planned and appropriate for experimental design.
- ❑ Manuscript text contains statement about institutional approval of a study (including IRB and IACUC protocol numbers), as well as adherence to guidelines on the treatment of animals and human subjects.

Results

- ❑ Results are presented in a logical, systematic fashion.
- ❑ Values of each measured variable are stated with error limits and statistical significance.

Conclusions

- ❑ The reported findings are interpreted and related to the stated hypothesis, as well as placed in clinical or physiologic perspective.

- ❑ Conclusion is succinct and confined to the study being reported, and avoids reference to other unrelated studies.
- ❑ The conclusion cites and briefly addresses all limitations of the current study.
- ❑ The authors refrain from imputing significance when statistical assessment does not reach the level of significance.
- ❑ For a clinical study, the conclusions emphasize how the findings might influence patient management or outcome.
- ❑ For a laboratory study, the conclusions suggest how findings shed light on the understanding of biologic processes and disease mechanisms.

Author Contributions

- ❑ The substantive contributions of all authors are accounted for in a short Author Contributions statement at the end of the text. Authors must fulfill all three of the following criteria:

  (i) each author must make substantial contributions to conception and design, acquisition of data, or analysis and interpretation of data

  (ii) each author must participate in drafting the article or critically revising it for intellectual content

  (iii) each author must give final approval of the version to be published.

References and Figures

- ❑ Original Articles, Current Opinions, and Special Reports contain no more than 40 references.
- ❑ Review Articles and Guidelines contain no more than 100 references.
- ❑ Procedures and Techniques and Brief Reports contain no more than 20 references.
- ❑ Figures are high-quality and enhance understanding of the discussed topic.
- ❑ Figures legends are easy to read and clearly labeled.
- ❑ Tables are clearly annotated with conventional symbols for statistical significance.

Contact Us

Interested in becoming a *J Trauma Acute Care Surg* reviewer? Please send a note of interest and your CV to the editor at info@jtrauma.org.
